# Association between lipid accumulation product and the prevalence of peripheral artery disease: A cross-sectional study based on NHANES 1999‒2004

**DOI:** 10.1016/j.clinsp.2026.101175

**Published:** 2026-09-28

**Authors:** Ling-Bin Mi, A-Bing Li

**Affiliations:** Department of Orthopedics, Ningbo Hospital of Integrated Traditional Chinese and Western Medicine, Ningbo, China

**Keywords:** Ankle-brachial index, Peripheral artery disease, Lipid accumulation product, National health and nutrition examination survey

## Abstract

**Background:**

This study aimed to evaluate the association between Lipid Accumulation Product (LAP) and the prevalence of Peripheral Artery Disease (PAD) among US adults using nationally representative data from the National Health and Nutrition Examination Survey (NHANES).

**Methods:**

Data from NHANES conducted between 1999 and 2004 were utilized for this analysis. Survey-weighted logistic regression models were used to examine the association between Log-transformed LAP (Ln-LAP) and PAD prevalence, with Ln-LAP analyzed both as a continuous variable and according to tertiles. Restricted cubic spline analysis was performed to assess the dose–response relationship. Prespecified subgroup analyses and exploratory machine-learning analyses using random forest and Light Gradient Boosting Machine models were also conducted.

**Results:**

Among 5,202 participants, 287 had PAD. The findings indicate a significant positive association between the natural logarithm of LAP (Ln-LAP) and the prevalence of PAD. After adjustment for potential confounders, each one-unit increase in Ln-LAP was associated with 36% higher odds of prevalent PAD (OR = 1.36, 95% CI 1.18–1.58). Notably, this association is particularly pronounced in specific subgroups, including women, individuals with estimated Glomerular Filtration Rate (eGFR) less than 60 mL/min/1.73 m^2^, and those who were high school graduates or above. In exploratory machine-learning analyses, Ln-LAP showed relatively high feature importance for PAD classification.

**Conclusion:**

Our study suggests a notable positive association between LAP levels and the prevalence of PAD. The association appeared to be approximately linear and may be more pronounced in specific subgroups. Given the cross-sectional design, causal inference cannot be made.

## Highlights

• Higher lipid accumulation product was associated with increased PAD prevalence.
• Ln-LAP showed a positive and nonlinear association with peripheral artery disease.
• The highest Ln-LAP tertile had a 51% higher PAD prevalence than the lowest tertile.
• Sex, eGFR, and education level modified the association between Ln-LAP and PAD.

## Introduction

Peripheral Artery Disease (PAD) is a chronic atherosclerotic vascular disorder that most commonly involves the lower extremity arteries. Patients with PAD may present with intermittent claudication, lower-limb pain, reduced exercise tolerance, and, in advanced cases, ulceration, tissue necrosis, chronic limb-threatening ischemia, or amputation. Beyond limb-related adverse outcomes, PAD is widely regarded as a clinical manifestation of systemic atherosclerosis and is closely associated with increased risks of coronary heart disease, stroke, and all-cause mortality.^1^ With population aging and the increasing burden of diabetes, hypertension, smoking, dyslipidemia, and obesity, PAD has become an increasingly important public health concern. Epidemiological evidence indicates that approximately 236 million adults worldwide were affected by PAD in 2015.^2^

Current risk assessment for PAD largely relies on traditional risk factors, including age, smoking, diabetes, hypertension, dyslipidemia, and prior cardiovascular disease.^3^ However, the development and progression of PAD involve multiple pathophysiological processes, including metabolic dysregulation, chronic inflammation, oxidative stress, endothelial dysfunction, and vascular remodeling. Traditional risk factors alone may therefore be insufficient to fully capture individual metabolic and vascular injury profiles.^3^ Accordingly, identifying simple and readily available markers that reflect metabolic abnormalities may help refine risk stratification and improve understanding of the cardiometabolic risk spectrum associated with PAD.

The Lipid Accumulation Product (LAP) is a sex-specific composite index calculated from waist circumference and triglyceride concentration. It was originally proposed by Kahn as a marker of central lipid accumulation and related cardiometabolic risk.^4^ Compared with Body Mass Index (BMI), which mainly reflects general adiposity, LAP incorporates information on both abdominal obesity and lipid metabolism and may therefore better reflect visceral fat accumulation and associated metabolic disturbances.^4^^,^^5^ Previous studies have shown that higher LAP levels are associated with insulin resistance, incident cardiovascular disease, and all-cause mortality.^5^, ^6^, ^7^

From a biological perspective, a plausible link may exist between LAP and PAD. Central adiposity and elevated triglyceride levels may contribute to atherosclerosis by promoting insulin resistance, chronic low-grade inflammation, oxidative stress, and endothelial dysfunction.^3^^,^^7^^,^^8^ These mechanisms are also central to the development and progression of PAD. Thus, LAP, as a simple marker of abdominal lipid accumulation and metabolic dysfunction, may be associated with the presence of PAD. However, epidemiological evidence on the relationship between LAP and PAD prevalence remains limited, particularly in nationally representative populations with adequate adjustment for demographic characteristics, lifestyle factors, and cardiometabolic conditions.

The National Health and Nutrition Examination Survey (NHANES) uses a complex, multistage probability sampling design and integrates questionnaire data, physical examinations, and laboratory measurements, providing an important data source for studying the health status of the noninstitutionalized US population.^9^ The 1999‒2004 NHANES cycles included Ankle-Brachial Index (ABI) measurements among adults aged 40-years and older, thereby providing a valuable basis for population-based PAD research.^10^ Although cross-sectional studies cannot establish temporal sequence or support causal inference, they can characterize epidemiological associations and generate hypotheses for future prospective and mechanistic investigations.

Therefore, using data from NHANES 1999‒2004, we aimed to evaluate the association between LAP and PAD prevalence among US adults aged 40-years or older with available ABI measurements. We further examined the dose-response pattern and assessed whether the association was consistent across prespecified subgroups.

## Methods

### Study design and data source

This cross-sectional study analyzed data from NHANES 1999‒2004. NHANES is a nationally representative survey of the noninstitutionalized US population and uses a complex, multistage, stratified probability sampling design. The survey consists of questionnaire interviews, physical examinations, and laboratory assessments.^9^ All NHANES protocols were reviewed and approved by the National Center for Health Statistics Research Ethics Review Board. Written informed consent was obtained from all participants before data collection. As the present study was a secondary analysis of publicly available, anonymized, and de-identified NHANES data, it was exempt from additional institutional review board approval, and no further informed consent was required. This cross-sectional study was conducted and reported according to the Strengthening the Reporting of Observational Studies in Epidemiology (STROBE) Statement guidelines.

The 1999‒2004 NHANES cycles initially included 31,126 participants. Participants were excluded if they had missing data required for LAP calculation, unavailable ABI data, age younger than 40-years, or variables with a missing proportion greater than 10% in the corresponding analyses. A total of 5,202 participants were included in the final analytic sample.

### Ankle-brachial index and definition of peripheral artery disease

ABI measurements were obtained from the NHANES Lower Extremity Disease examination component. Participants were examined in the supine position. Systolic blood pressure was measured at the brachial artery and at both ankles. According to the NHANES protocol, ankle pressures were measured at the posterior tibial arteries of both legs; the anterior tibial or dorsalis pedis arteries were not routinely assessed. Brachial pressure was measured in the right arm by default, and the left arm was used only when the right arm was unsuitable for measurement. For participants aged 40–59 years, systolic blood pressure was measured twice at each site, whereas for participants aged 60-years or older, one measurement was obtained at each site. The right and left ABI values were calculated as the mean systolic pressure of the corresponding ankle divided by the mean brachial systolic pressure. PAD was defined as an ABI ≤ 0.90 in either leg.^1^^,^^10^

### Lipid accumulation product

LAP was calculated using the sex-specific formulas originally proposed by Kahn:[4] LAP = [waist circumference (cm) - 65] × triglyceride concentration (mmol/L) for men, and LAP = [waist circumference (cm) - 58] × triglyceride concentration (mmol/L) for women.

Because LAP was right-skewed, it was natural log-transformed before analysis and expressed as Ln-LAP. Ln-LAP was analyzed both as a continuous variable and as tertiles. Tertile cut points were calculated according to the unweighted distribution of Ln-LAP and were defined as < 3.56, 3.56‒4.23, and > 4.23.

### Covariates

Potential confounders were selected according to previous literature and their clinical relevance to both metabolic status and PAD.^1^^,^^3^ The primary fully adjusted model included age, sex, race, educational level, smoking status, PIR, physical activity, alcohol consumption, eGFR, hypertension, CVD, and diabetes mellitus. Hypercholesterolemia, medication-use variables, and dietary supplement variables were considered in additional sensitivity analyses.

Age was modeled as a continuous variable and was categorized as ≤ 60 and > 60 years in subgroup analyses. Sex was categorized as male or female. Race/ethnicity was classified according to NHANES categories. Educational level was classified according to the highest level of education attained. PIR was categorized as below the poverty line, at or above the poverty line, and high income. Smoking status was categorized as never, former, or current smoking. Alcohol consumption was categorized as nondrinking, light drinking, or moderate drinking. Physical activity was categorized as sedentary, light-intensity, moderate-intensity, or vigorous-intensity activity. eGFR was categorized as ≥ 90, 60‒89, or < 60 mL/min/1.73 m^2^. Cardiovascular Disease (CVD) was defined as a self-reported history of physician-diagnosed congestive heart failure, coronary heart disease, angina/angina pectoris, heart attack/myocardial infarction, or stroke, based on the NHANES Medical Conditions Questionnaire. Hypercholesterolemia was defined as serum total cholesterol ≥240 mg/dL or current use of cholesterol-lowering medication. Hypertension and diabetes mellitus were defined using self-reported physician diagnosis, medication use, examination data, or laboratory data when available. Lipid-lowering medication use, antihypertensive medication use, and antidiabetic medication or insulin use were identified based on NHANES questionnaire data.

Because NHANES did not provide a dedicated variable specifically classifying nutraceutical use, dietary supplement use was used as a proxy measure. Lipid-related dietary supplement use was further identified based on reported supplement names potentially related to lipid metabolism or cardiovascular risk.

### Survey weighting and missing data

All analyses incorporated the NHANES complex sampling design, including sampling weights, strata, and primary sampling units, in accordance with NHANES analytic guidelines.^9^ Because triglyceride concentrations were measured in the fasting laboratory subsample, fasting subsample weights were applied. For the combined 1999‒2004 dataset, 6-year fasting subsample weights were calculated by dividing the corresponding 2-year fasting subsample weights by 3, consistent with NHANES recommendations for combining multiple survey cycles.^9^

Participants with missing exposure variables required for LAP calculation, missing ABI data, or missing covariates required for the primary analysis were excluded. For covariates with less than 10% missingness, multiple imputation was performed using the mice package in R.^11^ Variables with more than 10% missingness were excluded from the corresponding analyses according to the prespecified missing-data strategy.

### Statistical analysis

All statistical analyses were performed using R/RStudio version 4.2.1. The distributions of the two continuous variables, age and LAP, were assessed by visual inspection of histograms and Q-Q plots. LAP showed an evidently skewed distribution; therefore, both continuous variables were summarized as medians with interquartile ranges to ensure consistent presentation of baseline characteristics and to avoid assuming normality. Categorical variables were presented as numbers and weighted percentages. Baseline characteristics were compared between participants with and without PAD using survey-weighted statistical tests. Survey-weighted analyses were conducted to account for the complex sampling design of NHANES.^9^^,^^12^

Survey-weighted logistic regression models were used to evaluate the association between Ln-LAP and PAD prevalence, and Odds Ratios (ORs) with 95% Confidence Intervals (95% CIs) were calculated. Ln-LAP was analyzed both as a continuous variable and as a categorical variable according to tertiles. Three models were constructed: Non‐adjusted model; Model I was adjusted for age, sex, and race; and Model II was further adjusted for educational level, smoking status, PIR, physical activity, alcohol consumption, eGFR, hypertension, CVD, and diabetes mellitus. To evaluate the robustness of the main findings and to address the potential influence of pharmacological background, hypercholesterolemia, and dietary supplement use, additional sensitivity analyses were performed. In the first sensitivity model, the fully adjusted model was further adjusted for hypercholesterolemia, antihypertensive medication use, antidiabetic medication or insulin use, and any dietary supplement use. In the second sensitivity model, lipid-related dietary supplement use was included instead of any dietary supplement use. These analyses were conducted to determine whether the association between Ln-LAP and PAD prevalence remained consistent after accounting for these additional cardiometabolic and nutraceutical-related factors.

Trend tests across Ln-LAP tertiles were performed by modeling the tertile variable as an ordinal variable. Restricted Cubic Spline (RCS) analysis was used to assess the dose-response relationship between Ln-LAP and PAD prevalence. RCS models with three knots located at the 10^th^, 50^th^, and 90^th^ percentiles of Ln-LAP were fitted within the fully adjusted survey-weighted logistic regression model, and tests for overall association and nonlinearity were performed.^13^

Subgroup analyses were conducted using the fully adjusted model to examine whether the association between Ln-LAP and PAD differed across prespecified subgroups. Interaction effects were assessed by including multiplicative interaction terms between Ln-LAP and subgroup variables in the model. A two-sided p-value < 0.05 was considered statistically significant.

### Machine-learning analysis

Random Forest (RF) and Light Gradient Boosting Machine (LightGBM) models were used as exploratory analyses to evaluate the relative predictive contribution of candidate variables for PAD classification. RF is an ensemble learning method based on multiple decision trees, whereas LightGBM is an efficient gradient boosting decision-tree framework suitable for classification and feature-importance analysis.^14^^,^^15^ In this study, PAD status was treated as the binary outcome, and Ln-LAP, together with demographic, lifestyle, socioeconomic, renal function, and cardiometabolic variables, was included as candidate predictors.

The RF and LightGBM models were fitted using unweighted data. NHANES complex sampling weights were incorporated in the descriptive analyses and survey-weighted regression models, which served as the main analyses for population-level inference. In contrast, the machine-learning models were applied primarily for exploratory predictive performance assessment and variable-importance evaluation, rather than for estimating nationally representative associations.

Feature importance derived from the RF and LightGBM models was used to assess the relative predictive contribution of each variable within the models. These machine-learning analyses were considered exploratory and were not used to infer causality. Accordingly, variables with high feature importance were interpreted as having greater predictive contribution within the model rather than as determinants or causal risk factors for PAD.

## Results

### Baseline characteristics

The NHANES study conducted between 1999 and 2004 included a total of 31,126 participants. Among these, 21,156 individuals under the age of 40 were excluded from the analysis. Additionally, participants with incomplete bilateral ABI data (n = 2,399) or non-LAP values (n = 338) were also omitted. Furthermore, due to the excessive missing data regarding alcohol consumption, which exceeded 10%, an additional 2,031 individuals were excluded (Fig. 1). Statistical comparisons revealed significant differences between the groups concerning age, educational level, Ln-LAP, PIR, physical activity levels, smoking status, alcohol consumption, eGFR, hypertension, CVD, and diabetes mellitus (p < 0.05). Conversely, the distribution of demographic characteristics such as sex and racial groups did not differ significantly (p > 0.05). Compared with participants without PAD, those with PAD had a higher prevalence of hypercholesterolemia (47.74% vs. 34.43%, p < 0.001), lipid-lowering medication use (31.36% vs. 16.52%, p < 0.001), antihypertensive medication use (49.83% vs. 29.54%, p < 0.001), and antidiabetic medication or insulin use (21.25% vs. 8.26%, p < 0.001). In contrast, lipid-related dietary supplement use was similar between participants with and without PAD (27.87% vs. 28.26%, p = 0.888), as was any dietary supplement use (58.54% vs. 57.40%, p = 0.704). Comprehensive details can be found in Table 1.

**Fig. 1:**
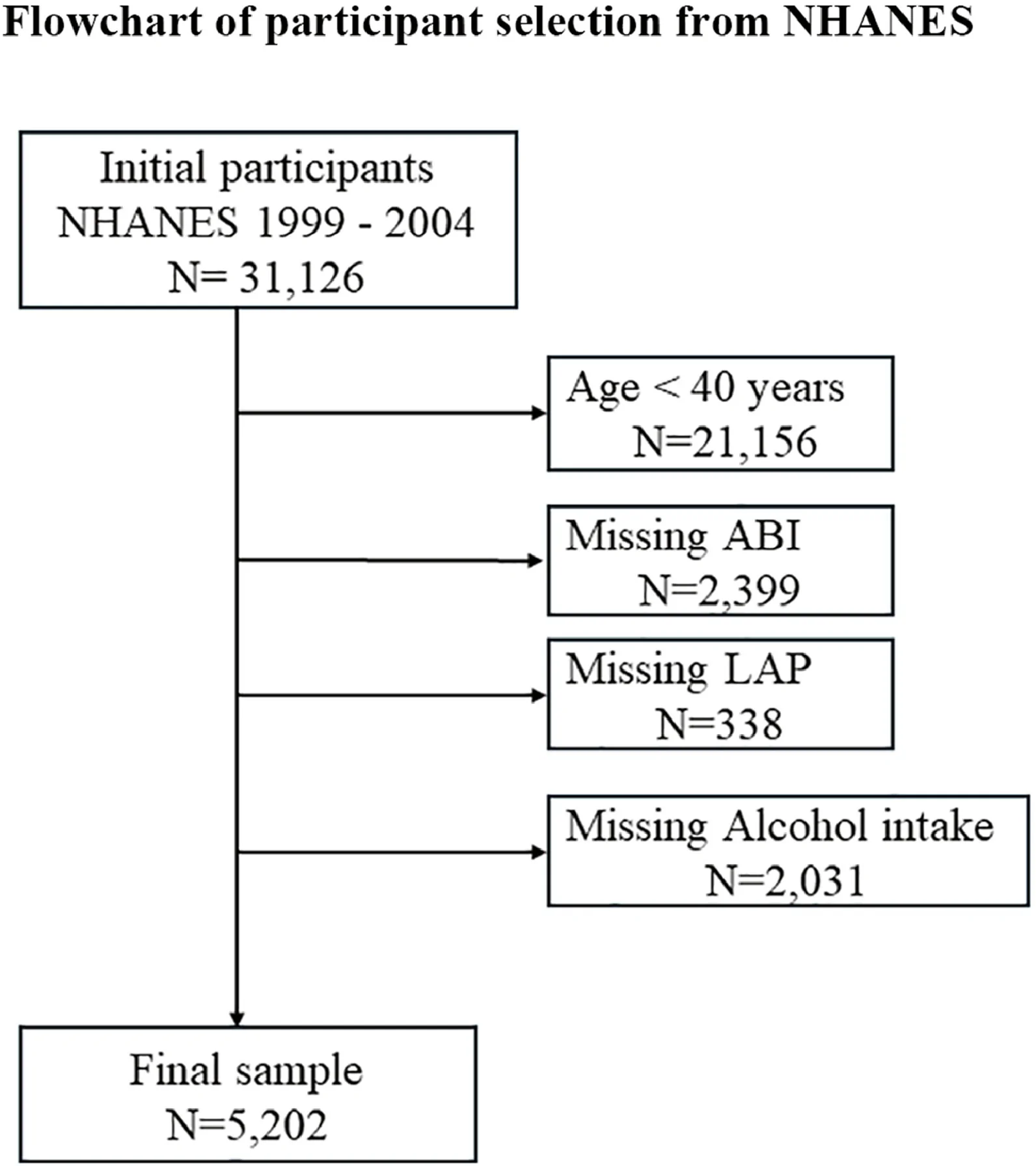
**Flowchart of participant selection from NHANES.** This flowchart illustrates the process used to identify the final analytic sample from the NHANES 1999–2004 cycles. Participants were excluded according to age eligibility, missing LAP data, missing ABI measurements, and missing covariate information. The final study population was used to evaluate the association between LAP and the prevalence of PAD. NHANES, National Health and Nutrition Examination Survey; LAP, Lipid Accumulation Product; ABI, Ankle-Brachial Index; PAD, Peripheral Artery Disease.

**Table 1:** Baseline characteristics of the study population.

Characteristics	Overall (n = 5202)	Without PAD (n = 4915)	With PAD (n = 287)	p-value
Age median [IQR] (year)	59.00 (40.00‒85.00)	57.00 (40.00‒85.00)	72.00 (43.00‒85.00)	**<0.001**
LAP median [IQR]	50.00 (1.50‒1515.67)	49.76 (1.50‒1515.67)	57.11 (4.80‒803.93)	**0.001**
Ln-LAP median [IQR]	3.91 (0.40‒7.32)	3.91 (0.40‒7.32)	4.04 (1.57‒6.69)	**0.001**
**Sex (%)**				0.614
Male	2812 (54.06%)	2661 (54.14%)	151 (52.61%)	
Female	2390 (45.94%)	2254 (45.86%)	136 (47.39%)	
**Race (%)**				0.13
Mexican American	1238 (23.80%)	1185 (24.11%)	53 (18.47%)	
White	3012 (57.90%)	2835 (57.68%)	177 (61.67%)	
Black	813 (15.63%)	762 (15.50%)	51 (17.77%)	
Other	139 (2.67%)	133 (2.71%)	6 (2.09%)	
**Education level (%)**				**<0.001**
Less than High School	1521 (29.24%)	1402 (28.52%)	119 (41.46%)	
High School Graduate/GED	1229 (23.63%)	1161 (23.62%)	68 (23.69%)	
More Than High School	2452 (47.14%)	2352 (47.85%)	100 (34.84%)	
**PIR (%)**				**<0.001**
Below Poverty Line	632 (12.15%)	585 (11.90%)	47 (16.38%)	
At or Above Poverty Line	3401 (65.38%)	3196 (65.03%)	205 (71.43%)	
High Income	1169 (22.47%)	1134 (23.07%)	35 (12.20%)	
**Hypertension (%)**				**<0.001**
No	2481 (47.69%)	2411 (49.05%)	70 (24.39%)	
Yes	2721 (52.31%)	2504 (50.95%)	217 (75.61%)	
CVD				**<0.001**
No	4570 (87.85%)	4364 (88.79%)	206 (71.78%)	
Yes	632 (12.15%)	551 (11.21%)	81 (28.22%)	
**Smoking status (%)**				**<0.001**
Never smoker	2229 (42.85%)	2144 (43.62%)	85 (29.62%)	
Current smoker	1131 (21.74%)	1050 (21.36%)	81 (28.22%)	
Former smoker	1842 (35.41%)	1721 (35.02%)	121 (42.16%)	
**Physical activity (%)**				**<0.001**
Light-intensity	1236 (23.76%)	1138 (23.15%)	98 (34.15%)	
Sedentary behaviors	2908 (55.90%)	2757 (56.09%)	151 (52.61%)	
Moderate/vigorous	1058 (20.34%)	1020 (20.75%)	38 (13.24%)	
**Alcohol intake (%)**				**0.004**
Non-drinker	754 (14.49%)	692 (14.08%)	62 (21.60%)	
Light-drinker	895 (17.20%)	847 (17.23%)	48 (16.72%)	
Moderate-drinker	2462 (47.33%)	2335 (47.51%)	127 (44.25%)	
High-drinker	1091 (20.97%)	1041 (21.18%)	50 (17.42%)	
**eGFR (%)**				**<0.001**
< 60	552 (10.61%)	472 (9.60%)	80 (27.87%)	
60–89	2087 (40.12%)	1951 (39.69%)	136 (47.39%)	
≥ 90	2563 (49.27%)	2492 (50.70%)	71 (24.74%)	
**Diabetes (%)**				**<0.001**
No	4338 (83.39%)	4138 (84.19%)	200 (69.69%)	
Yes	864 (16.61%)	777 (15.81%)	87 (30.31%)	
**Ln-LAP tertile**				**0.033**
< 3.56	1734 (33.33%)	1655 (33.67%)	79 (27.53%)	
3.56–4.23	1734 (33.33%)	1640 (33.37%)	94 (32.75%)	
> 4.23	1734 (33.33%)	1620 (32.96%)	114 (39.72%)	
**Age categorical (%)**				**<0.001**
< 60	2650 (50.94%)	2609 (53.08%)	41 (14.29%)	
≥ 60	2552 (49.06%)	2306 (46.92%)	246 (85.71%)	
**Hypercholesterolemia (%)**				**<0.001**
No	3373 (64.84%)	3223 (65.57%)	150 (52.26%)	
Yes	1829 (35.16%)	1692 (34.43%)	137 (47.74%)	
**Lipid-lowering medication use (%)**				**<0.001**
No	4300 (82.66%)	4103 (83.48%)	197 (68.64%)	
Yes	902 (17.34%)	812 (16.52%)	90 (31.36%)	
**Antihypertensive medication use (%)**				**<0.001**
No	3607 (69.34%)	3463 (70.46%)	144 (50.17%)	
Yes	1595 (30.66%)	1452 (29.54%)	143 (49.83%)	
**Antidiabetic medication or insulin use (%)**				**<0.001**
No	4735 (91.02%)	4509 (91.74%)	226 (78.75%)	
Yes	467 (8.98%)	406 (8.26%)	61 (21.25%)	
**Lipid-related dietary supplement use (%)**				0.888
No	3733 (71.76%)	3526 (71.74%)	207 (72.13%)	
Yes	1469 (28.24%)	1389 (28.26%)	80 (27.87%)	
**Any dietary supplement use (%)**				0.704
No	2213 (42.54%)	2094 (42.60%)	119 (41.46%)	
Yes	2989 (57.46%	2821 (57.40%)	168 (58.54%)	

Data are presented as median [IQR] or n (%). Statistically significant p-values (p < 0.05) are shown in bold. IQR, Interquartile Range; PAD, Peripheral Artery Disease; PIR, Poverty-Income Ratio; CVD, Cardiovascular Disease; eGFR, estimated Glomerular Filtration Rate; LAP, Lipid Accumulation Product; Ln-LAP, Natural Logarithm-transformed Lipid Accumulation Product.

PIR was classified as follows: below poverty line, PIR < 1; at or above poverty line, 1 ≤ PIR < 5; and high income, PIR ≥ 5. Missing data for variables with less than 10% missing values were handled using multiple imputation with the mice package in R.

### Association between Ln-LAP and PAD

To address the right-skewed distribution of LAP data, a logarithmic transformation (Ln-LAP) was employed. The outcomes of a logistic regression analysis assessing the relationship between the prevalence of PAD and Ln-LAP are summarized in Table 2. The results consistently illustrated a significant positive association between Ln-LAP and PAD risk across unadjusted models (OR = 1.53, 95% CI 1.35–1.74), models adjusted for age, sex, and ethnicity (OR = 1.63, 95% CI 1.41–1.88), and fully adjusted models (OR = 1.36, 95% CI 1.18–1.58). Notably, after comprehensive adjustment for covariates, a significant positive association persisted between the highest tertile of Ln-LAP levels and PAD prevalence (OR = 1.51, 95% CI 1.11–2.05). Subsequent analyses converting Ln-LAP into a categorical variable indicated a significant upward trend in PAD prevalence with rising Ln-LAP levels (p-trend < 0.001).

**Table 2:** Association between the Ln-LAP and the prevalence of peripheral artery disease.

Variables	Non‐adjusted model		Model I		Model II	
	OR (95% CI)	p-value	OR (95% CI)	p-value	OR (95% CI)	p-value
Ln-LAP	1.53 (1.35, 1.74)	<0.0001	1.63 (1.41, 1.88)	<0.0001	1.36 (1.18, 1.58)	0.0004
Ln-LAP (Tertiles)						
< 3.56	Reference		Reference		Reference	
3.56–4.23	1.33 (0.89, 1.99)	0.1692	1.15 (0.75, 1.75)	0.5313	1.02 (0.68, 1.52)	0.9217
> 4.23	2.09 (1.55, 2.81)	<0.0001	2.05 (1.54, 2.72)	0.0004	1.51 (1.11, 2.05)	0.0156
p trend		<0.0001		<0.0001		0.0128

Data are presented as the estimated value with its 95% Confidence Interval.

Non-adjusted model: No variables were adjusted.

Model I: Adjusted for age, race, and sex.

Model II: Adjusted for age, sex, race, education level, PIR smoking status, alcohol intake, eGFR, CVD, physical activity, diabetes, and hypertension.

Ln-LAP, Natural Logarithm Lipid Accumulation Product; PIR, Poverty-Income Ratio; CVD, Cardiovascular Diseases; eGFR, Estimated Glomerular Filtration Rate; OR, Odds Ratio; CI, Confidence Interval.

### Sensitivity analyses

As shown in Table 3, the association between Ln-LAP and PAD prevalence remained generally consistent after further adjustment for hypercholesterolemia, antihypertensive medication use, antidiabetic medication or insulin use, and any dietary supplement use (OR = 1.35, 95% CI 1.15–1.58). Similar results were observed when lipid-related dietary supplement use was included instead of any dietary supplement use (OR = 1.34, 95% CI 1.14–1.59). These findings suggest that the observed association between Ln-LAP and PAD prevalence was not substantially explained by pharmacological background, hypercholesterolemia, diabetes-related treatment, or dietary supplement use.

**Table 3:** Sensitivity analyses of the association between Ln-LAP and peripheral artery disease prevalence.

Variables	Model II		Sensitivity Model I		Sensitivity Model II	
	OR (95% CI)	p-value	OR (95% CI)	p-value	OR (95% CI)	p-value
Ln-LAP	1.36 (1.18, 1.58)	0.0004	1.35 (1.15, 1.58)	0.0021	1.34 (1.14, 1.59)	0.0028
Ln-LAP (Tertiles)						
< 3.56	Reference		Reference		Reference	
3.56–4.23	1.02 (0.68, 1.52)	0.9217	1.01 (0.68, 1.52)	0.9500	1.00 (0.67, 1.51)	0.9864
> 4.23	1.51 (1.11, 2.05)	0.0156	1.50 (1.08, 2.07)	0.0285	1.49 (1.07, 2.07)	0.0322
p trend		0.0128		0.0246		0.0273

Data are presented as the estimated value with its 95% confidence interval.

Model II: Adjusted for age, sex, race, education level, PIR smoking status, alcohol intake, eGFR, CVD, physical activity, diabetes, and hypertension.

Sensitivity Model I: Adjusted for age, sex, race, education level, PIR smoking status, alcohol intake, eGFR, CVD, physical activity, diabetes, hypertension, hypercholesterolemia, antihypertensive medication use, antidiabetic medication or insulin use, and any dietary supplement use.

Sensitivity Model II: Adjusted for age, sex, race, education level, PIR smoking status, alcohol intake, eGFR, CVD, physical activity, diabetes, hypertension, hypercholesterolemia, antihypertensive medication use, antidiabetic medication or insulin use, and lipid-related dietary supplement use.

Ln-LAP, Natural Logarithm Lipid Accumulation Product; PIR, Poverty-Income Ratio; CVD, Cardiovascular Diseases; eGFR, Estimated Glomerular Filtration Rate; OR, Odds Ratio; CI, Confidence Interval.

### Restricted cubic splines

RCS were employed to explore the dose–response relationship between Ln-LAP and PAD prevalence. The RCS findings revealed a clear linear relationship between Ln-LAP and PAD prevalence (P for overall = 0.016, P for non-linearity = 0.766) (Fig. 2).

**Fig. 2:**
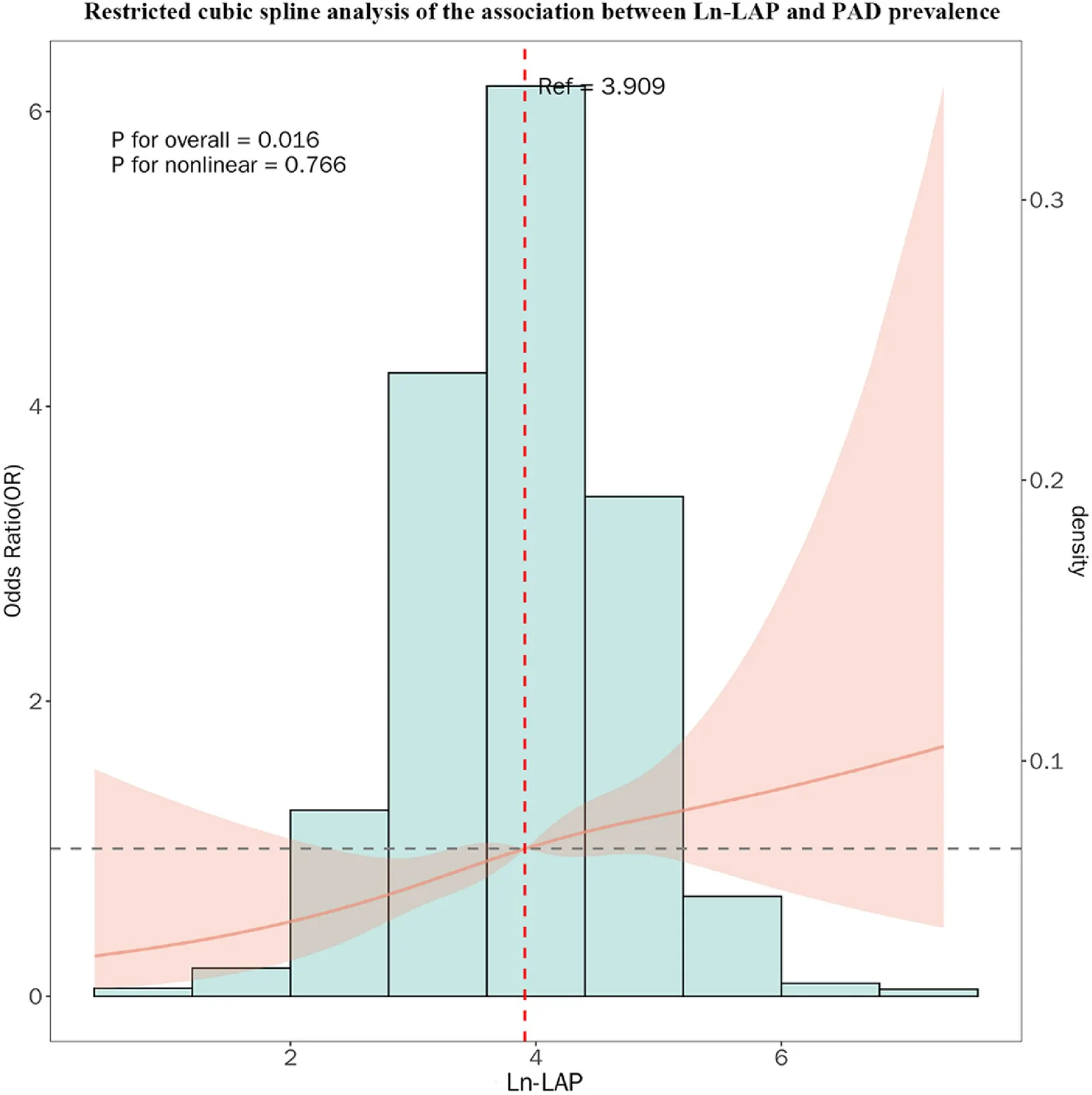
**Restricted cubic spline analysis of the association between Ln-LAP and PAD prevalence.** The association between Ln-LAP and the prevalence of PAD was evaluated using RCS models within the fully adjusted survey-weighted logistic regression model. Three knots were placed at the 10^th^, 50^th^, and 90^th^ percentiles of Ln-LAP. The model was adjusted for age, sex, race, education level, PIR, smoking status, alcohol intake, eGFR, CVD, physical activity, diabetes, and hypertension. The solid curve represents the estimated OR for PAD across different levels of Ln-LAP, and the shaded area represents the 95% Confidence Interval (95% CI). The histogram shows the distribution of Ln-LAP, with density shown on the right y-axis. The vertical dashed line indicates the reference value of Ln-LAP, set at 3.909, and the horizontal dashed line at OR = 1 indicates no association. Tests for overall association and nonlinearity were performed. Ln-LAP, Natural Logarithm-transformed Lipid Accumulation Product; PAD, Peripheral Artery Disease; RCS, Restricted Cubic Spline; PIR, Poverty-Income Ratio; eGFR, Estimated Glomerular Filtration Rate; CVD, Cardiovascular Disease; OR, Odds Ratio; CI, Confidence Interval.

### Subgroup analysis

We conducted a subgroup analysis utilizing Model 3 (Fig. 3) in conjunction with stratification variables, including age, sex, race, physical activity, smoking status, diabetes, eGFR, and hypertension, to investigate potential associations between Ln-LAP and PAD. The results indicated a consistent positive association between the prevalence of PAD and Ln-LAP levels.

**Fig. 3:**
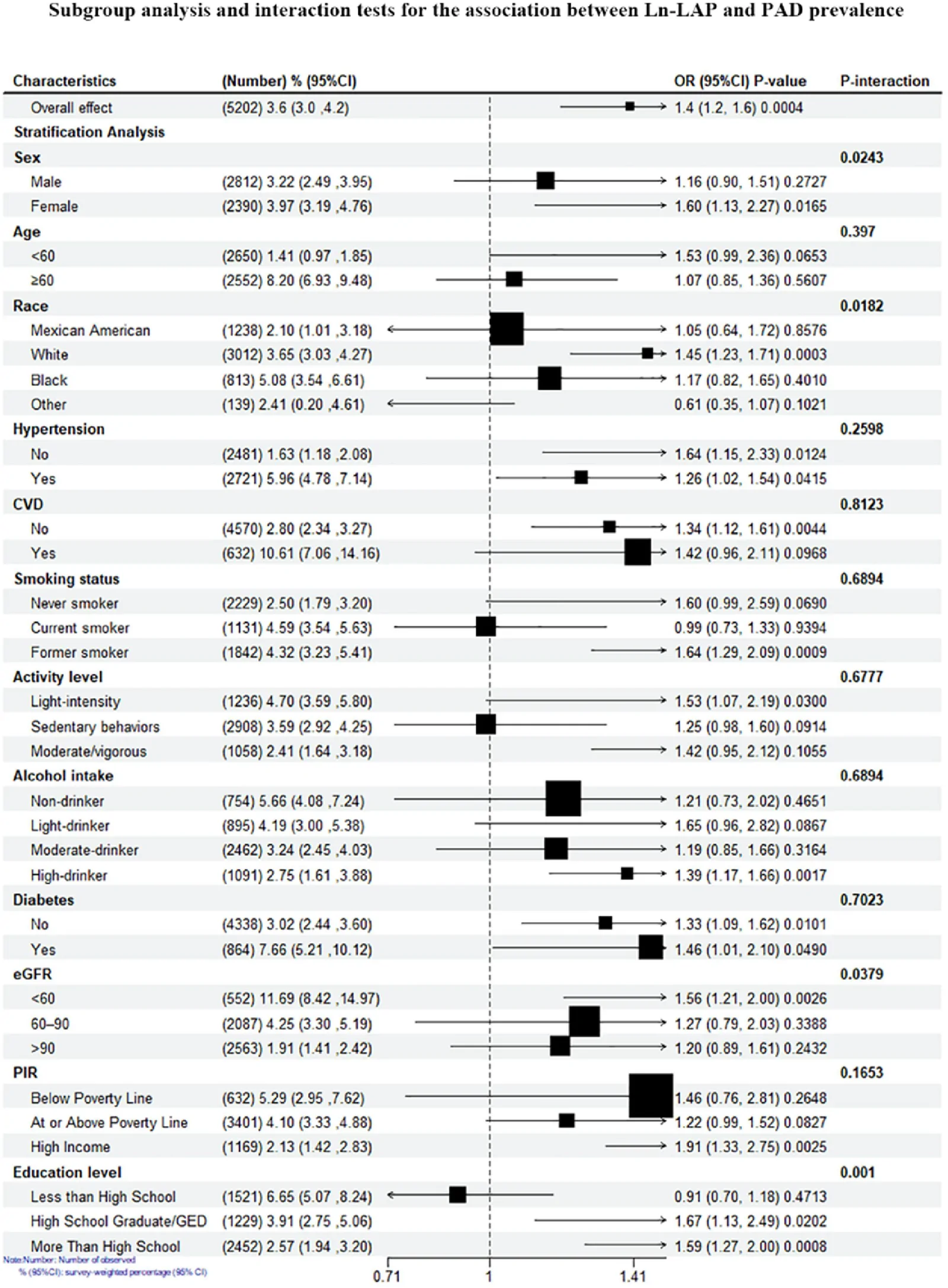
**Subgroup analysis and interaction tests for the association between Ln-LAP and PAD prevalence.** Forest plot showing the association between Ln-LAP and the prevalence of PAD across predefined subgroups. Survey-weighted PAD prevalence is presented as percentage with 95% Confidence Interval. Odds Ratio (OR) and 95% Confidence Interval (95% CI) were calculated using survey-weighted logistic regression models. The vertical dashed line indicates an OR of 1.0. p-values indicate subgroup-specific associations, and P for interaction was used to assess whether the association between Ln-LAP and PAD differed across strata. The models were adjusted for age, sex, race, education level, PIR, smoking status, alcohol intake, eGFR, CVD, physical activity, diabetes, and hypertension, as appropriate. Interaction tests were performed by including product terms between Ln-LAP and each subgroup variable. Ln-LAP, Natural Logarithm-transformed Lipid Accumulation Product; PAD, Peripheral Artery Disease; PIR, Poverty-Income Ratio; eGFR, Estimated Glomerular Filtration Rate; CVD, Cardiovascular Disease; OR, Odds Ratio; CI, Confidence Interval.

Our findings suggested that the significant association between Ln-LAP and PAD persisted across various subgroups, specifically among female participants, high alcohol consumers, individuals of white, those with a poverty income ratio of 5 or higher, high school graduates or individuals with higher education, former smokers, individuals with eGFR below 60, as well as those with and without diabetes and hypertension. Interaction tests further suggested that the positive relationship between Ln-LAP and PAD prevalence was particularly pronounced in females (P for interaction = 0.0243), individuals with eGFR less than 60 (P for interaction = 0.0379), and those who were high school graduates or above (P for interaction = 0.001).

### Machine learning

The feature importance plot derived from the RF regression model emphasizes the relative significance of various clinical and demographic variables in predicting PAD. This plot distinctly suggests that clinical parameters associated with fat distribution and lipid metabolism rank among the most critical predictors of PAD, with the Ln-LAP serving as a significant factor in the model and a pivotal indicator in this investigation (Fig. 4A). Additionally, we employed the lightGBM model to analyze these variables, yielding results consistent with those obtained from the random forest analysis, thereby reinforcing the notion that the Ln-LAP showed relatively high feature importance in both machine-learning models (Fig. 4B).

**Fig. 4:**
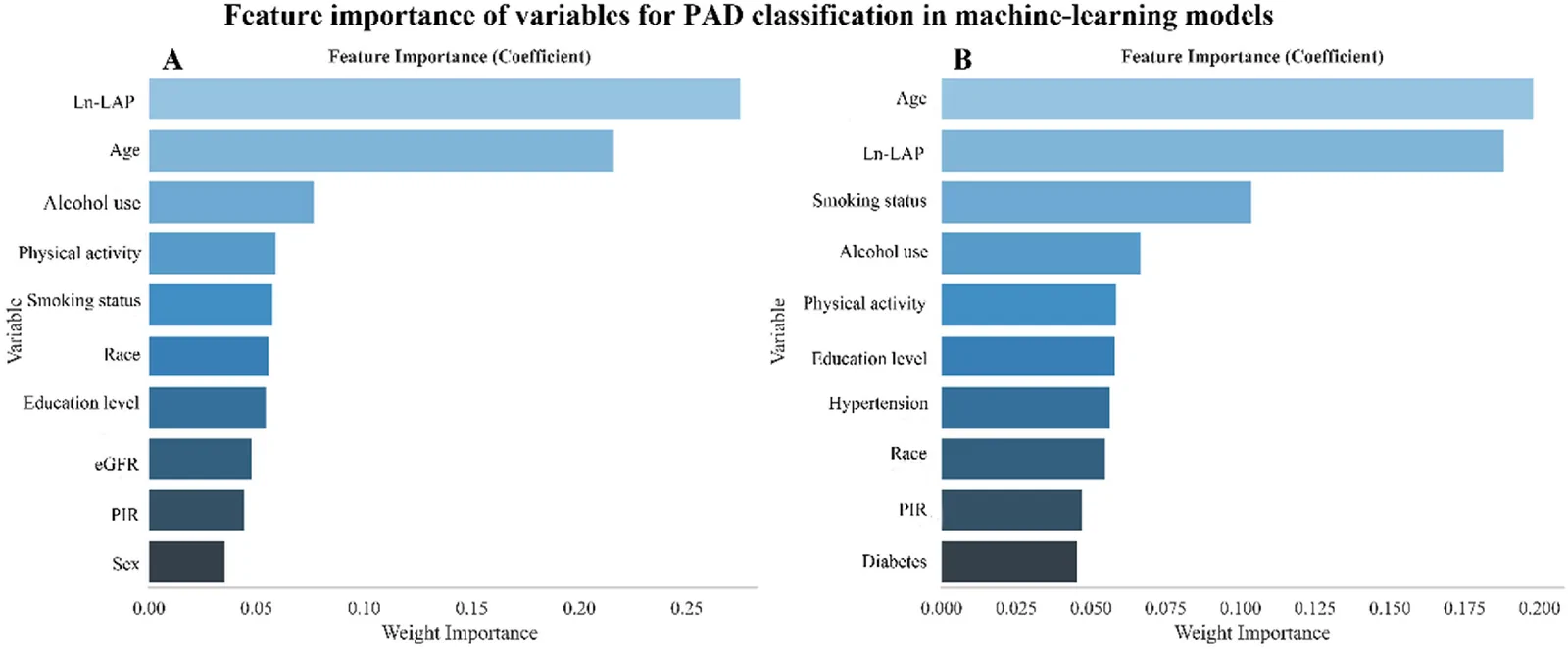
**Feature importance of variables for PAD classification in machine-learning models.** Bar plots showing the relative importance of variables for PAD classification in two machine-learning models. Random Forest model (A) and Light Gradient Boosting Machine model (B) represent the feature-importance rankings derived from the two models used in the analysis. The machine-learning models were fitted using unweighted data and were applied as exploratory predictive analyses. Bar length represents the relative feature importance of each variable within the corresponding model. Ln-LAP, Natural Logarithm-transformed Lipid Accumulation Product; PAD, Peripheral Artery Disease; PIR, Poverty-Income Ratio; eGFR, Estimated Glomerular Filtration Rate.

## Discussion

Using data from NHANES 1999‒2004, this study systematically evaluated the association between LAP and PAD prevalence among US adults aged 40-years or older. We found that higher Ln-LAP was positively associated with PAD prevalence. This association persisted after adjustment for demographic characteristics, socioeconomic status, lifestyle factors, renal function, and cardiometabolic comorbidities. Compared with participants in the lowest Ln-LAP tertile, those in the highest tertile had a higher prevalence of PAD, with a statistically significant trend across tertiles. RCS analysis further suggested an approximately linear positive association between Ln-LAP and PAD prevalence. These findings indicate that LAP, a simple marker reflecting abdominal lipid accumulation and metabolic dysfunction, may be closely related to prevalent PAD. Given the cross-sectional design, however, these results should be interpreted as epidemiological associations rather than evidence that elevated Ln-LAP causes PAD.

LAP is calculated from waist circumference and triglyceride concentration and represents a sex-specific composite metabolic index. Unlike BMI and other conventional adiposity indices, LAP captures both central adiposity and lipid metabolic abnormalities and may therefore more sensitively reflect visceral fat accumulation and related metabolic dysregulation. Kahn first proposed LAP as an index of excessive lipid accumulation and cardiometabolic risk, and subsequent studies have linked higher LAP levels to insulin resistance, metabolic syndrome, incident cardiovascular disease, and all-cause mortality.^4^, ^5^, ^6^, ^7^ The present findings extend this evidence to PAD, suggesting that the lipid accumulation and metabolic disturbance captured by LAP may also be related to peripheral atherosclerotic burden.

Several biological pathways may underlie the observed association. First, visceral adiposity can increase free fatty acid release, insulin resistance, and ectopic lipid deposition, thereby aggravating endothelial dysfunction. Second, hypertriglyceridemia often coexists with triglyceride-rich lipoproteins and their remnants, small dense LDL particles, low HDL-C levels, and chronic low-grade inflammation. Triglyceride-rich lipoproteins and remnant particles may contribute to atherosclerosis by promoting vascular inflammation, endothelial dysfunction, and plaque progression.^16^ Third, obesity-related inflammation and oxidative stress can reduce nitric oxide bioavailability, impair vasodilation, and enhance vascular wall inflammation and lipid deposition.^8^^,^^17^ PAD is fundamentally a manifestation of systemic atherosclerosis in the peripheral arterial system, and its development and progression are closely linked to endothelial injury, inflammation, oxidative stress, and lipid accumulation.^1^^,^^3^ Thus, elevated Ln-LAP may not represent a single risk factor but rather a composite metabolic phenotype associated with prevalent PAD.

When Ln-LAP was analyzed according to tertiles, PAD prevalence increased across higher Ln-LAP categories. RCS analysis also indicated an approximately linear association between Ln-LAP and PAD prevalence, without evidence of a pronounced nonlinear or threshold effect. These results suggest that lipid accumulation-related metabolic abnormalities may be continuously associated with PAD burden in this population. Prior studies have reported linear, nonlinear, or saturation patterns between LAP and various clinical outcomes, including cardiovascular disease, chronic kidney disease, and abdominal aortic calcification, depending on the study population, outcome type, and metabolic context.^6^^,^^18^^,^^19^ The near-linear association observed in the present study suggests that LAP may be useful as a continuous marker of PAD-related metabolic abnormality rather than being clinically relevant only at very high values. Nevertheless, prospective cohort studies are needed to confirm the temporal sequence and incremental predictive value of LAP for PAD.

Subgroup analysis suggested that the positive association between Ln-LAP and PAD prevalence was more evident among women, with a statistically significant interaction. Importantly, sex itself was not significantly associated with PAD prevalence in the baseline characteristics shown in Table 1. Therefore, this finding should not be interpreted as evidence of a higher overall PAD prevalence in women; rather, it suggests that the linear association between Ln-LAP and PAD prevalence may be particularly pronounced in the female subgroup. This observation is broadly consistent with prior evidence indicating sex-related differences in PAD. Previous studies have shown that women with PAD may differ from men in risk factor profiles, symptom presentation, diagnostic delay, functional limitation, and treatment outcomes.^20^^,^^21^ Reviews by Pabon et al. and Kim et al. emphasized that sex differences in PAD extend beyond prevalence and involve vascular biology, clinical presentation, and management patterns.^20^^,^^21^

From a metabolic perspective, changes in fat distribution and lipid metabolism across the female life course may modify the relationship between LAP and PAD. Premenopausal women may have estrogen-related vascular protection, whereas declining estrogen levels and the menopausal transition are accompanied by unfavorable changes in body fat distribution, lipid and lipoprotein profiles, and vascular health.^22^ These changes are closely related to the two components of LAP, namely waist circumference and triglyceride concentration. Therefore, particularly among middle-aged and older women, LAP may capture the combined atherosclerotic burden related to fat redistribution and lipid metabolic disturbance. In this context, LAP may reflect not only adiposity but also postmenopausal metabolic transition, dyslipidemia, and vascular dysfunction.

In addition, women with PAD often present with atypical symptoms, more pronounced functional limitation, or delayed diagnosis.^20^^,^^21^ These characteristics may weaken the explanatory capacity of traditional risk factors for prevalent PAD in women. A composite metabolic index such as LAP may therefore have greater relevance for identifying prevalent PAD among women. However, this study did not include information on menopausal status, hormone replacement therapy, pregnancy-related complications, or other female-specific cardiovascular risk factors. Therefore, this subgroup finding should be considered exploratory and hypothesis-generating rather than evidence of a definitive sex-specific mechanism.

The association between Ln-LAP and PAD prevalence also appeared stronger among participants with a high school education or above. This finding is complex and should be interpreted cautiously. Prior analyses of NHANES 1999‒2004 reported that lower income and lower educational level were generally associated with higher PAD prevalence. Socioeconomic determinants may also influence the development and care of PAD through health behaviors, living environments, access to care, and biological pathways.^23^^,^^24^ Therefore, the stronger association observed in participants with higher educational attainment should not be interpreted as indicating that higher education itself increases PAD risk. A more appropriate interpretation is that educational level may modify the observed strength of the LAP-PAD association rather than directly alter the biological mechanism of PAD.

Several explanations are possible. First, participants with higher educational attainment may have greater access to health examinations and disease detection, increasing the probability that PAD is identified and thereby strengthening the association between LAP and detected PAD. Second, occupational activity, sedentary behavior, diet, health literacy, and medication adherence may differ across educational strata and may influence the relationship between LAP and PAD. Third, among participants with higher educational attainment, the influence of some socioeconomic disadvantage-related factors may be less prominent, allowing the metabolic information captured by LAP to be more clearly expressed in the model. Conversely, among participants with lower educational attainment, smoking, limited access to care, occupational exposures, and suboptimal chronic disease management may have stronger effects and could attenuate or obscure the LAP-PAD association. These explanations are consistent with the multidimensional and complex roles of socioeconomic determinants in PAD development and care.^24^ Finally, subgroup analyses involve multiple comparisons, and the interaction by educational level may represent a chance finding.

Thus, rather than attributing this result to psychological stress associated with higher education, a more cautious interpretation is that the positive subgroup finding for educational level may reflect heterogeneity in socioeconomic context and health behaviors. Because this direction is not fully consistent with prior evidence, further validation is required. In the absence of direct measures of psychological stress, occupational burden, cortisol levels, or related pathways, overinterpretation of this subgroup finding should be avoided.

In the present study, participants with PAD had a substantially higher burden of metabolic, cardiovascular, and renal comorbidities than those without PAD. Diabetes, hypertension, CVD, reduced eGFR, and hypercholesterolemia were all more prevalent in the PAD group. These findings indicate that PAD is not an isolated lower-extremity vascular disorder, but rather occurs in the context of systemic cardiometabolic and renal comorbidity.

Diabetes is a particularly important metabolic risk factor for PAD. Chronic hyperglycemia, insulin resistance, oxidative stress, advanced glycation end-product accumulation, endothelial dysfunction, vascular inflammation, platelet activation, and impaired vascular repair can jointly accelerate lower-extremity atherosclerosis and arterial stiffness.^25^^,^^26^ Diabetes-related neuropathy and microvascular injury may also mask typical claudication symptoms, delay PAD diagnosis, and increase the risk of foot ulceration, chronic limb-threatening ischemia, impaired wound healing, infection, and amputation.^27^^,^^28^ In our subgroup analysis, the association between Ln-LAP and PAD remained significant in both non-diabetic participants [OR = 1.33, 95% CI: 1.09–1.62] and diabetic participants [OR = 1.46, 95% CI: 1.01–2.10]. Although the interaction between diabetes status and Ln-LAP was not statistically significant, the slightly higher point estimate in the diabetic subgroup suggests that diabetes represents an important high-risk metabolic background in which lipid accumulation, inflammation, and vascular injury may coexist. Therefore, our findings support the need to pay particular attention to diabetes status when evaluating PAD risk, especially in individuals with elevated LAP or Ln-LAP.

Renal dysfunction also deserves attention. In our subgroup analysis, the association between Ln-LAP and PAD appeared stronger among participants with eGFR < 60 mL/min/1.73 m^2^ [OR = 1.56, 95% CI: 1.21–2.00]. This finding is biologically plausible because eGFR < 60 mL/min/1.73 m^2^ generally indicates chronic kidney disease or clinically relevant renal dysfunction, which is closely linked to PAD and adverse cardiovascular and limb outcomes.^29^^,^^30^ Chronic kidney disease may increase PAD risk through the accumulation of traditional cardiovascular risk factors, chronic inflammation, oxidative stress, endothelial dysfunction, vascular calcification, and mineral metabolism abnormalities.^29^^,^^31^ In addition, renal dysfunction is frequently accompanied by dyslipidemia, including elevated triglycerides, impaired clearance of triglyceride-rich lipoproteins, and increased remnant lipoproteins, all of which may promote atherosclerosis.^32^ Therefore, the coexistence of elevated LAP and reduced eGFR may indicate a high-burden state characterized by concurrent metabolic dysfunction, renal impairment, and vascular injury. Previous evidence also suggests that LAP is associated with chronic kidney disease risk, supporting the concept that LAP may reflect a composite metabolic and renal risk profile.^19^

Other comorbidities may also contribute to PAD through overlapping vascular mechanisms. Hypertension can promote arterial stiffness, endothelial dysfunction, vascular remodeling, and atherosclerotic progression. CVD reflects systemic atherosclerosis and may indicate a polyvascular disease phenotype involving coronary, cerebrovascular, and lower-extremity arteries. Hypercholesterolemia contributes to lipid deposition, plaque formation, vascular inflammation, and arterial stenosis, thereby accelerating lower-extremity atherosclerotic disease.^30^^,^^33^ Consistent with these mechanisms, our subgroup analysis showed that the positive association between Ln-LAP and PAD was generally observed across different comorbidity profiles.

In our study, participants with PAD had higher use of lipid-lowering medications, antihypertensive medications, and antidiabetic medications or insulin than those without PAD. These findings are consistent with the higher prevalence of hypercholesterolemia, hypertension, and diabetes in the PAD group, suggesting that participants with PAD had a greater cardiometabolic treatment burden. In addition, these medications may influence lipid metabolism, blood pressure control, glucose homeostasis, vascular inflammation, endothelial function, and atherosclerotic progression. Therefore, pharmacological background is an important factor to consider when interpreting the association between Ln-LAP and PAD.

To further evaluate whether medication use influenced our findings, we performed additional sensitivity analyses by incorporating medication-use variables into the fully adjusted model. The association between Ln-LAP and PAD remained materially unchanged after additional adjustment, supporting the robustness of our main findings.

Taken together, these findings suggest that diabetes, hypertension, CVD, renal dysfunction, and hypercholesterolemia may jointly contribute to PAD development and progression. Therefore, the relationship between Ln-LAP and PAD should be interpreted within the broader context of cardiometabolic and renal multimorbidity.

Scicchitano et al. discussed the potential role of nutraceuticals and functional food ingredients in dyslipidemia management and cardiovascular prevention, suggesting that some compounds may have favorable effects on lipid metabolism, oxidative stress, inflammation, endothelial function, and vascular health.^34^ Recent reviews have also suggested that several nutraceuticals may influence lipid profiles and atherosclerosis-related pathways, including cholesterol metabolism, triglyceride metabolism, oxidative stress, endothelial dysfunction, and vascular inflammation.^35^^,^^36^ However, the current evidence remains heterogeneous, and there is still limited evidence that nutraceuticals improve clinically meaningful cardiovascular outcomes; evidence regarding PAD-specific limb-related outcomes, such as chronic limb-threatening ischemia, amputation, and mortality, is even more limited.^36^^,^^37^ In the present study, NHANES did not provide a dedicated variable specifically identifying nutraceuticals or functional food ingredients; therefore, any dietary supplement use and lipid-related dietary supplement use were used as available proxy indicators. These supplement-related variables were similar between participants with and without PAD, and additional adjustment for these variables did not materially change the association between Ln-LAP and PAD. Therefore, although nutraceuticals may represent a complementary strategy for cardiometabolic risk modification, they should not be interpreted as a substitute for guideline-directed pharmacological therapy or comprehensive vascular risk management.

The machine-learning analyses showed that Ln-LAP had relatively high feature importance in both the RF and LightGBM models. This result was directionally consistent with the conventional regression analyses and suggests that Ln-LAP may provide predictive information for identifying prevalent PAD. Compared with single adiposity measures, LAP integrates abdominal obesity and triglyceride concentration and may more comprehensively reflect the metabolic abnormalities relevant to atherosclerosis. Nevertheless, feature importance in machine-learning models represents relative predictive contribution within a given model and does not establish causality or identify determinants of PAD. According to reporting guidance such as TRIPOD+AI, model development and validation studies should adequately report discrimination, calibration, validation strategy, and transparency.^38^ Therefore, in the absence of complete model performance assessment, cross-validation, and external validation, the machine-learning analyses should be viewed as exploratory supplementary evidence rather than evidence that a clinical prediction model has been established.

This study has several strengths. First, NHANES uses a complex, multistage probability sampling design and, with appropriate weighting, can represent the noninstitutionalized US adult population. Second, PAD was defined using ABI, which is more objective than self-reported disease history alone. Third, the association between Ln-LAP and PAD prevalence was assessed from multiple perspectives, including continuous-variable analysis, tertile-based analysis, RCS modeling, and subgroup analyses. Fourth, the RF and LightGBM analyses provided additional evidence from the perspective of predictive contribution.

Several limitations should also be acknowledged. First, because of the cross-sectional design, this study cannot determine whether elevated Ln-LAP preceded PAD and therefore cannot support causal inference. Second, NHANES did not provide a dedicated variable specifically classifying nutraceutical or functional food use. Dietary supplement use during the past 30-days was therefore used as a proxy measure for nutraceutical exposure, but the available data could not fully capture specific product formulation, dosage, duration of use, adherence, or functional food intake. Residual confounding related to nutraceuticals and dietary patterns therefore cannot be completely excluded. Third, LAP depends on waist circumference and fasting triglyceride concentration, and triglycerides were obtained from the NHANES fasting laboratory subsample; sample selection and weight handling may therefore influence the estimates. Fourth, subgroup analyses involved multiple comparisons, and some subgroups may have had limited numbers of events; interaction results should therefore be interpreted cautiously. Fifth, the machine-learning analysis models did not incorporate the complex NHANES sampling weights. Therefore, the machine-learning findings should be interpreted as exploratory predictive analyses rather than nationally representative inferential estimates.

## Conclusion

In conclusion, among US adults aged 40-years or older, higher Ln-LAP was independently associated with a higher prevalence of PAD, and the association appeared approximately linear in RCS analysis. Subgroup analyses suggested that this association may be more pronounced among women, participants with eGFR < 60 mL/min/1.73 m^2^, and those with a high school education or above. Given the cross-sectional design, causal inference cannot be made. Prospective cohort studies, mechanistic investigations, and externally validated models are needed to clarify the potential value of LAP for PAD risk identification and clinical stratification.

## Consent for publication

Not applicable.

## Clinical trial number

Not applicable.

## Declaration of generative AI and AI-assisted technologies

During the preparation of this work, the author(s) used Grammarly to check grammar and enhance language clarity. After using this tool, the author(s) reviewed and edited the content as needed and take(s) full responsibility for the content of the publication.

## Funding

The medical and health research project of Zhejiang province (2023RC268) and Zhejiang Provincial Traditional Chinese Medicine Science and Technology Program Project (2025ZX016).

## Data availability statement

Data available from the corresponding author upon reasonable request.

## Declaration of competing interest

The authors declare no have conflicts of interest.
